# Advanced Paternal Age Does Not Affect Medically-Relevant Obstetrical and Perinatal Outcomes following IVF or ICSI in Humans with Donated Oocytes

**DOI:** 10.3390/jcm12031014

**Published:** 2023-01-28

**Authors:** Ana Navarro-Gomezlechon, María Gil Juliá, Irene Hervás, Laura Mossetti, Rocío Rivera-Egea, Nicolás Garrido

**Affiliations:** 1 IVI Foundation—Instituto de Investigación Sanitaria La Fe (IIS La Fe), Av. Fernando Abril Martorell, 106, Torre A, 46026 Valencia, Spain; 2 IVF Laboratory, IVIRMA Roma, Via Federico Calabresi, 11, 00169 Rome, Italy; 3 Andrology Laboratory and Sperm Bank, IVIRMA Valencia, Plaza de la Policia Local 3, 46015 Valencia, Spain

**Keywords:** paternal age, assisted reproductive technology (ART), obstetrical outcomes, perinatal outcomes, donated oocytes, pregnancy, offspring’s health

## Abstract

Background: Concomitant with delays in childbearing, concerns have been raised of whether advanced paternal age is associated with adverse reproductive outcomes, but the evidence is controversial in part due to the uncertain threshold in which to consider advanced paternal age and confounding maternal factors. This retrospective study aimed to evaluate the effect of paternal age on reproductive outcomes related to the pregnancy and perinatal health of the offspring. Methods: We retrospectively evaluated 16,268 cases of patients who underwent IVF or ICSI (using autologous sperm and donated oocytes, between January 2008 and March 2020, at Spanish IVIRMA clinics. Patients were divided based on paternal age at conception [≤30 (*n* = 204), 31–40 (*n* = 5752), and >40 years (*n* = 10,312)], and the differences in obstetrical and perinatal outcomes were analyzed by descriptive analysis, followed by univariate and multivariate analysis. Results: Fathers 31–40 and >40 years old were associated with lower odds of caesarean delivery [AOR 0.63 (95% CI, 0.44–0.90; *p* = 0.012) and AOR 0.61 (95% CI, 0.41–0.91; *p* = 0.017), respectively] and longer pregnancies [ARC 5.09 (95% CI, 2.39–7.79; *p* < 0.001) and ARC 4.54 (95% CI, 1.51–7.58; *p* = 0.003), respectively] with respect to fathers ≤30 years old. Furthermore, fathers aged 31–40 years old had lower odds of having a female infant (AOR, 0.70; 95% CI, 0.49–0.99; *p* = 0.045) than those ≤30. The rest of obstetrical and perinatal outcomes, which we deemed more medically-relevant as they were considered serious for health, were comparable between groups with our adjusted model. Conclusions: Despite this hopeful message to fathers of advanced paternal age, future studies should consider the short- and long-term outcomes of the offspring and try to better elucidate the associations of advanced paternal age with reproductive outcomes and the molecular mechanisms underlying the observed associations.

## 1. Introduction

In recent years, delays in childbearing have increased the average maternal and paternal age at which the first child is conceived [1,2]. These delays are due to various sociocultural factors including educational, professional, economic, and personal changes, increased life expectancy, improved contraception, advanced age at marriage, and the availability of assisted reproductive technologies (ARTs). In this regard, studying the possible effect(s) of age on reproductive outcomes is becoming increasingly relevant [3,4,5,6,7,8,9].

While the influence of advanced maternal age (>35 years) on reproductive outcomes, pregnancy, and offspring health has been extensively characterized [10,11,12,13], it has been difficult to establish a similar cut-off in men (although it has been proposed as >40 [14,15,16]), since the evidence on the effects of advanced paternal age is currently limited. With the considerable reduction in fertility and elevated chromosomal aneuploidy (notably augmenting the risk of Down syndrome, among other disorders) correlated with advanced maternal age, concerns have been raised of whether advanced paternal age may also be associated with adverse reproductive outcomes, or potential obstetrical or perinatal risks.

Among the few studies that have evaluated the consequence(s) of advanced paternal age on male fertility potential, reproductive success, pregnancy, and offspring health, the findings are controversial, and the study designs were not appropriate [5,6,17,18,19,20]. Although some studies have not found associations [21,22], others have agreed that advanced paternal age affects reproductive hormones, testicular function, and spermatogenesis, altering clinical semen parameters (measured with basic semen analysis), molecular markers related to fertility (e.g., reactive oxygen species, telomeres, and DNA integrity), and offspring genetics (through aneuploidy, epigenetics, and de novo mutations), ultimately resulting in infertility and/or adverse reproductive outcomes [3,5,14,19,23,24,25]. Recently, several studies have pointed out the potential involvement of the increase in paternal age in a wide range of adverse outcomes related to the pregnancy and health of the offspring. In this regard, advanced paternal age has been associated with an increased risk of spontaneous miscarriage [15], stillbirth [26], premature birth, low birth weight [27,28], low Apgar score [29], gestational diabetes, and caesarean section [14,24]. However, not all studies have found such associations between any of these variables and increased paternal age, so the results are controversial [20,30,31]. Offspring pathologies that have been associated with advanced paternal age include several cancers (e.g., pediatric brain cancers, retinoblastoma, acute lymphoblastic leukemia, and non-Hodgkin lymphoma [32,33]; and adult breast, prostate, and nervous system cancers [34]), orofacial clefts (i.e., cleft lip and palate) [14,23,34], achondroplasia [26], and Apert syndrome [35], along with congenital heart defects [34,36,37]. Indeed, Fang et al. found that compared to fathers aged 25–29, fathers ≥40 years old could increase the risk of cardiovascular abnormalities, facial deformities, urogenital abnormalities, and chromosome disorders in the offspring [38]. Additionally, the prevalence of Down syndrome, autism spectrum disorders [39], schizophrenia [40], and bipolar disorders [41] is also postulated to be augmented in association with advanced paternal age [14]. Finally, while some studies have found an increase in embryo chromosomal aneuploidy with advanced paternal age [42,43], others have not [6,7]. Taken together, this evidence suggests that paternal age could be associated with reproductive risks related to pregnancy and offspring health, however, further research is necessary.

As advanced paternal age is often accompanied by advanced maternal age, which may also contribute to negative obstetric and perinatal outcomes [12], studies using donated oocytes can, to some extent, standardize and homogenize the female factors [44,45] to more confidently study the effects of the male factors. In this regard, two novelties of this current study were the use of donated oocytes and the consideration of maternal age in the adjusted analysis to more confidently evaluate the possible association(s) of paternal age with greater risks of problems related to the pregnancy and offspring health.

The present study aimed to evaluate the effect of paternal age on the reproductive outcomes related to the pregnancy and offspring health in couples undergoing in vitro fertilization (IVF) or intracytoplasmic sperm injection (ICSI) using donated oocytes and autologous sperm in a large population of patients in order to control the female contribution to the main outcomes evaluated.

## 2. Materials and Methods

### 2.1. Study Design

This retrospective, observational, multicentric cohort study evaluated the reproductive outcomes of couples that underwent at least one IVF or ICSI cycle (using the father’s own sperm and donated oocytes) between January 2008 and March 2020 at a Spanish IVIRMA clinic, and had clinical follow-up during and after the pregnancy. Cases in which the semen samples were obtained from testicular biopsy or epididymis aspirate were excluded. We also excluded IVF/ICSI cycles in which half of the oocytes were inseminated by IVF and the other half by ICSI. Only singleton deliveries were included, and only the first delivery of each patient was considered. We included couples when a pregnancy was achieved whether they had a live birth or not.

Patients included in the study had different female and male etiologies for infertility, or did not have any. Teratozoospermia, oligozoospermia, or karyotype alteration were some of the male etiologies for infertility. Regarding female etiologies, some of them included karyotype alteration, endometriosis, low ovarian reserve, maternal age, premature ovarian failure, or polycystic ovarian syndrome.

Relevant clinical outcomes were extracted from the electronic medical records of the patients, and compiled into a database to filter erroneous or incomplete data and analyze the study variables.

### 2.2. Assisted Reproductive Technologies

Ejaculated semen samples were liquefied for 30 min at 37 °C and 5% CO_2_, and standard semen analysis was used to evaluate several macroscopic (i.e., volume, pH, and viscosity) and microscopic parameters (i.e., concentration, motility, and morphology). Sperm was capacitated using the swim-up technique [46] or density gradients [47].

Oocyte donors and recipients underwent controlled ovarian stimulation and endometrial preparation, respectively, as previously described [48,49]. Oocytes were retrieved from donors, decumulated, and inseminated by conventional IVF or ICSI [50]. The resulting embryos were cultured, and embryo development was evaluated [47]. If clinically indicated, embryos were biopsied for preimplantation genetic testing (PGT) [51]. Finally, embryos were transferred, and a clinical follow-up was conducted to assess the reproductive outcomes of the couple.

### 2.3. Outcome Measures

The outcome measures of the following study included several obstetrical and perinatal outcomes. In terms of obstetrical outcomes, we considered type of delivery (caesarean versus vaginal), preterm birth (<37 weeks), gestational diabetes, anemia, hypertension, pre-eclampsia (presence of hypertension and proteinuria after 20 weeks of gestation), and premature rupture of membranes (PROM; before week 37). Regarding the perinatal outcomes, we evaluated the neonate’s gestational age, sex, weight (low birth weight was defined as <2500 g), length, cranial perimeter, Apgar score (1, 5, and 10 min), and admission to the neonatal intensive care unit (NICU). We also measured the gestational results in terms of fetal death, perinatal death, live birth, and premature live birth. Data export was conducted to obtain the clinical database followed by the filtering of the data and the statistical analysis.

### 2.4. Statistical Analysis

We first conducted a descriptive analysis, followed by univariate and multivariate model analysis using the youngest group of men (≤30 years) as a reference for the models. In the descriptive analysis, ANOVAs were used to compare the continuous variables, while Chi-squares were employed for the categorical variables. For the univariate model analysis, generalized linear models were applied for the categorical variables and linear models were applied for the continuous variables. Multivariate analysis was performed adjusting for maternal age, maternal body mass index (BMI), paternal age, fresh sperm sample concentration and progressive motility, insemination technique, cycle type, gestational age, transfer on day 5, and type of delivery (when appropriate).

All analyses were carried out in R (version 4.0.3). In all cases, *p* < 0.05 was considered statistically significant.

## 3. Results

### 3.1. Baseline Patient and ART Characteristics

A total of 16,268 couples (with fathers aged 21–54 years old) were included in the study. Patients were arbitrarily divided into three groups, based on paternal age at conception [≤30 (*n* = 204), 31–40 (*n* = 5752), and >40 years old (*n* = 10,312)]. The clinical characteristics of the participants in each group including the patient, cycle, and semen characteristics are presented in Table 1. Patients included in the study had different female and male etiologies for infertility, or did not have any.

### 3.2. Association of Paternal Age and Obstetrical Outcomes

Paternal age (i.e., ≤30, 31–40, and >40 years old) had no significant effect on the comparison of gestational results between groups in terms of fetal death [0.00% (95% CI, 0.00–1.79), 0.03% (95% CI, 0.00–0.13), and 0.07% (95% CI, 0.03–0.14), respectively], perinatal death [0.00% (95% CI, 0.00–1.79), 0.14% (95% CI, 0.06–0.27), and 0.07% (95% CI, 0.03–0.14), respectively], live birth [97.55% (95% CI, 94.37–99.20), 99.04% (95% CI, 98.76–99.28), and 99.02% (95% CI, 98.81–99.20), respectively], or premature live birth [2.45% (95% CI, 0.80–5.63), 0.78% (95% CI, 0.57–1.05), and 0.84% (95% CI, 0.68–1.04), respectively] over the total number of pregnancies.

Significant differences were found between the age groups in terms of gestational diabetes, hypertension, pre-eclampsia, PROM, and type of delivery, but not for anemia or preterm birth (Table 2).

The partners of men ≤30 years old had a significantly increased risk of developing hypertension than the partners of men aged 31–40 (OR, 0.40; 95% CI, 0.23–0.70; *p* = 0.001) or >40 years old (OR, 0.50; 95% CI, 0.29–0.86; *p* = 0.013); experiencing preeclampsia than partners of men 31–40 years old (OR, 0.38; 95% CI, 0.15–0.98; *p* = 0.045); PROM than the partners of men 31–40 (OR, 0.37; 95% CI, 0.17–0.78; *p* = 0.009) or >40 years old (OR, 0.35; 95% CI, 0.17–0.74; *p* = 0.006); and having a preterm birth than the partners of men >40 years old (OR, 0.68; 95% CI, 0.46–1.00; *p* = 0.047). Furthermore, fathers >40 years old were associated with a significantly increased risk of caesarean delivery (OR, 1.98; 95% CI, 1.48–2.65; *p* < 0.001) (Table 2).

Given the retrospective nature of our study and the possibility to have relevant variables related to the outcomes differently distributed among groups, to avoid potential biases derived from the difference between the groups, the statistical differences we observed among our groups (Table 1) were accounted for in subsequent statistical modeling. Specifically, we adjusted for maternal age and BMI, paternal age, fresh sperm sample concentration and progressive motility, insemination technique, cycle type, gestational age, embryo transfer on day 5, and type of delivery (when appropriate). Considering these potential confounders, paternal age did not significantly affect gestational diabetes, anemia, hypertension, preeclampsia, PROM, or preterm birth. However, paternal age ≤30 years old was found to significantly increase the risk of having a caesarean delivery, with respect to fathers that were 31–40 (AOR, 0.63; 95% CI, 0.44–0.90; *p* = 0.012) and >40 years old (AOR, 0.61; 95% CI, 0.41–0.91; *p* = 0.017), assuming all other covariates were at the baseline (Table 2).

### 3.3. Association of Paternal Age and Perinatal Outcomes

Among the 16,244 deliveries that resulted in a live birth, 204 were from fathers ≤30 years old; 5742 were from fathers 31–40 years old; and 10,298 were from fathers >40 years old. Comparing the different age groups, we found significant differences related to gestational age (in days; *p* < 0.001), infant length (*p* = 0.010), and cranial perimeter (*p* = 0.012). However, paternal age was not significantly related to infant sex (*p* = 0.11), weight (*p* = 0.535), low birth weight (*p* = 0.279), NICU admission (*p* = 0.063), or Apgar score at 1, 5, and 10 min (*p* = 0.256, *p* = 0.478, and *p* = 0.112, respectively) (Table 3).

Using the group of fathers ≤30 years old as the reference, partners of fathers 31–40 years old had significantly longer gestations (RC 3.49; 95% CI, 1.23–5.74; *p* = 0.002), and infants born to fathers 31–40 and >40 years old were significantly longer (RC 0.82 [95% CI, 0.27–1.37; *p* = 0.003] and RC 0.84 [95% CI, 0.30–1.39; *p* = 0.002], respectively), and had significantly lower odds of being admitted to the NICU (OR 0.54 [95% CI, 0.31–0.92; *p* = 0.025] and OR 0.53 [95% CI, 0.31–0.91; *p* = 0.021], respectively). No significant differences were found among the remaining variables analyzed (Table 3). Notably, after adjusting for the potential confounding variables, the effect of paternal age on the duration of the pregnancy was even more pronounced, with an ARC of 5.09 (95% CI, 2.39–7.79; *p* < 0.001) for the fathers aged 31–40, and ARC of 4.54 (95% CI, 1.51–7.58; *p* = 0.003) for fathers >40. Fathers aged 31–40 had 30% lower odds of having a female infant (AOR, 0.70; 95% CI, 0.49–0.99; *p* = 0.045) than those ≤30. As for the remaining perinatal outcomes, no significant associations were found (Table 3).

## 4. Discussion

The present retrospective study aimed to evaluate the effect(s) of paternal age on obstetrical and perinatal outcomes, following IVF or ICSI using autologous sperm and donated oocytes, in a nationwide-population cohort. Using donated oocytes allowed us to adequately model male contributions to the reproductive outcomes by removing, to some extent, the female biases (e.g., advanced maternal age) [44,45]. Indeed, our findings demonstrated that maternal and paternal age were positively correlated (Table 1), but the mean oocyte donor age was comparable between all groups (24.95 for ≤30 vs. 25.44 for 31–40 vs. 25.42 for >40; *p* = 0.254).

While several studies have pointed out the potential involvement of the increase in paternal age in a wide range of adverse outcomes related to pregnancy and the offspring’s health [14,24,26,27,28,29], the evidence remains controversial [20,30,31]. Our study did initially find statistically significant differences for a few obstetrical and perinatal outcomes between men aged 31–40 and >40 with those ≤30 years. However after accounting for several confounding variables (i.e., maternal age and BMI, paternal age, fresh sperm sample concentration and progressive motility, insemination technique, cycle type, gestational age, embryo transfer on day 5, and type of delivery [when appropriate]), advanced paternal age at conception was only associated with a minor risk of having a caesarean delivery, a longer pregnancy, and lower odds of having a female infant, which, sometimes has more to do with personal rather than medical decisions, in the case of caesarean, or that have limited clinical relevance as for having a female newborn or having relatively higher gestational age. Indeed, the outcomes that we considered to have more medical relevance (as they were considered serious for the health of the mother or newborn) were found to be comparable by our adjusted model. Nevertheless, due to the evident delays in childbearing highlighted by this study (with 35.36% of participants between 31 and 40 and 63.39% >40 years old), the effects of advanced paternal age merit further evaluation with future prospective studies that consider the short- and/or long-term outcomes of the offspring, and preclinical models that try to elucidate the molecular mechanisms underlying the observed differences [5].

Regarding obstetrical complications, our initial comparisons revealed that anemia more than doubled when the paternal age was >40 rather than ≤30 (10.51% vs. 3.95%, respectively), which corresponded with the AOR of 3.43 (95% CI, 0.77–15.35) we found between these two groups, however, these results were not significant. Similarly, our initial comparisons revealed that the risk of gestational diabetes doubled with paternal age >40 compared to ≤30 (12.80% vs. 6.49%, respectively), and our univariate and multivariate models confirmed this finding with an OR of 2.11 (95% CI, 0.85–5.25) and an AOR of 1.04 (95% CI, 0.33–3.21), but there were no significant differences. Interestingly, Khandwala et al. also reported a higher OR of gestational diabetes when the father was aged older than 45 [14,24], however, their study did not involve ART nor oocyte donation. On the other hand, after accounting for maternal age and other risk factors, Hurley and DeFranco did not find that increased paternal age was associated with a significant increase in the rates of preeclampsia, preterm birth, or NICU admission [20,30,31], as was the case with our multivariate analysis.

Although our findings support those of Chen et al., in that advanced paternal age (>40 years) was not a risk factor for adverse outcomes in the offspring [20,30,31], there was a discord with other previous studies with regard to the perinatal outcomes. Specifically, we found no significant difference between the mean Apgar scores (1, 5, and 10) of infants born to fathers aged 31–40 or >40 years compared to ≤30, which contradicts a study from Sun et al., who found a modest effect of advanced paternal age on the Apgar score [29]. Furthermore, our univariate and multivariate analyses indicated that paternal age was not significantly associated with birth weight or low birth weight (<2500 g), in contrast to the study by Chung et al., who found that paternal age, among others, was significantly associated with low birth weight in the univariate and multivariate analysis [28], and to the study by Goisis et al., who also found an association between paternal age and low birth weight [27].

In some countries, advanced maternal age is a limitation for the access to, efficacy, and success of ART, but such limitations do not currently exist for men, mainly because there is no consensus on what is considered to be advanced paternal age, and the effects it may have on reproductive outcomes remain controversial. We acknowledge that the different cut-offs used for the paternal age classes in each study can influence the interpretation of the results and impede the discovery of possible associations. In this regard, establishing and standardizing a threshold age for men where fertility decreases and reproductive risks increase (comparable to the one established for women at 35 years of age) can dually aid in personal family planning and clinical decision-making, especially in the context of reproductive medicine counselling and fertility care. Moreover, it should also be considered if studies used oocyte donation or adjusted for maternal age to control for this important confounding factor. Furthermore, although several investigations have examined the possible association between the increase in paternal age and reproductive variables, there is still a need to better clarify the molecular mechanisms that can cause these associations [5].

Finally, due to the retrospective nature of this study, there were some clinical biases and there was some missing data (i.e., incomplete patient histories) limiting the sample size, but statistical power was still achieved by evaluating a nationwide-population cohort. It must be noticed that the major strength of this study was the use of oocyte donation standardizing female factors (to some extent).

## 5. Conclusions

Due to the evident delays in fatherhood, concerns have been raised of whether advanced paternal age can be associated with adverse reproductive outcomes, and studies focusing on this topic are increasing, although evidence on this matter remains controversial. Our study revealed some statistically significant associations between the increase in paternal age and obstetrical and perinatal outcomes following IVF or ICSI using autologous sperm and donated oocytes. Specifically, we found that fathers >30 years old were associated with a decreased risk of caesarean delivery and longer gestations, and fathers 31–40 years old had lower odds of having a female infant than men ≤30. Although these findings were interesting, we considered them to be of less clinical relevance than the other outcomes we evaluated, and within this context, our study sends a hopeful message to fathers of advanced paternal age. Nonetheless, future perspectives comprise the need for further well-defined preclinical and prospective clinical studies to respectively better elucidate the associations of advanced paternal age with reproductive outcomes and the molecular mechanisms underlying the observed associations, and improve reproductive counselling and fertility care. Finally, the consideration of oocyte donation treatments standardizing female factors (to some extent) and the large sample size should be emphasized as important points of the study.

## Figures and Tables

**Table 1:** Baseline characteristics and ART details of the study population.

	≤30	31–40	>40	*p*
Number of patients	204	5752	10,312	
Paternal age (years)	28.71 (28.48–28.95)	37.42 (37.36–37.48)	43.93 (43.88–43.97)	<0.001 *
Paternal BMI (kg/m^2^)	23.66 (23.10–24.22)	23.03 (22.93–23.12)	23.48 (23.41–23.56)	<0.001 *
Maternal age (years)	28.47 (28.21–28.72)	37.14 (37.08–37.21)	43.58 (43.54–43.63)	<0.001 *
Maternal BMI (kg/m^2^)	23.60 (23.06–24.14)	23.02 (22.93–23.12)	23.47 (23.40–23.54)	<0.001 *
Oocyte donor age (years)	24.95 (24.37–25.52)	25.44 (25.33–25.55)	25.42 (25.33–25.50)	0.254
Sperm concentration (million/mL)	47.06 (41.76–52.36)	42.66 (41.78–43.53)	44.29 (43.61–44.96)	0.006 *
Progressive motility of sperm	28.79 (25.35–32.23)	32.62 (32.04–33.21)	31.32 (30.90–31.74)	<0.001 *
Number of oocytes	13.10 (12.56–13.65)	12.96 (12.86–13.06)	12.78 (12.71–12.86)	0.013 *
Insemination technique				<0.001 *
IVF	8.82% (5.31–13.59)	3.46% (3.00–3.96)	3.44% (3.10–3.81)	
ICSI	91.18% (86.41–94.69)	96.54% (96.04–97.00)	96.56% (96.19–96.90)	
Oocyte state				<0.001 *
Fresh	51.60% (44.21–58.93)	57.92% (56.61–59.23)	55.10% (54.11–56.08)	
Vitrified	46.81% (39.51–54.21)	40.20% (38.90–41.50)	43.76% (42.78–44.74)	
Mixed	1.60% (0.33–4.59)	1.88% (1.54–2.27)	1.14% (0.94–1.37)	
Cycle type				<0.001 *
Stimulated	1.49% (0.31–4.28)	0.62% (0.43–0.86)	0.38% (0.27–0.52)	
Natural	4.46% (2.06–8.29)	8.22% (7.52–8.97)	6.22% (5.76–6.71)	
Substituted	94.06% (89.85–96.89)	91.16% (90.39–91.89)	93.39% (92.89–93.87)	
Capacitation method				0.019 *
Density gradient	51.47% (44.39–58.51)	56.43% (55.14–57.72)	56.74% (55.78–57.70)	
Swim-up	41.67% (34.82–48.76)	35.34% (34.11–36.60)	35.04% (34.12–35.97)	
Only washed	1.96% (0.54–4.94)	2.54% (2.15–2.98)	3.29% (2.95–3.65)	
Embryo transfer				<0.001 *
Prior to day 5	31.37% (25.07–38.22)	26.73% (25.59–27.89)	22.29% (21.49–23.10)	
On or after day 5	68.63% (61.78–74.93)	73.27% (72.11–74.41)	77.71% (76.90–78.51)	

Results are presented as a proportion (for categorical variables) or mean (for continuous variables) with corresponding 95% confidence intervals and *p* value of the comparisons between age groups. BMI, body mass index; ICSI, intracytoplasmic sperm injection; IVF, in vitro fertilization. * *p* < 0.05.

**Table 2:** Obstetrical outcomes associated with paternal age following in vitro fertilization or intracytoplasmic sperm injection using autologous sperm and donated oocytes.

	Proportion (95% CI)	*p*	OR (95% CI)	*p*	AOR (95% CI)	Adjusted *p*
Gestational diabetes		<0.001 *				
≤30	6.49% (2.14–14.51)		Reference	-	Reference	-
31–40	9.80% (8.68–11.01)		1.56 (0.63–3.91)	0.338	1.11 (0.38–3.23)	0.845
>40	12.80% (11.84–13.80)		2.11 (0.85–5.25)	0.107	1.04 (0.33–3.21)	0.953
Anemia		0.167				
≤30	3.95% (0.82–11.11)		Reference	-	Reference	-
31–40	10.70% (9.53–11.95)		2.92 (0.91–9.31)	0.071	3.47 (0.82–14.69)	0.092
>40	10.51% (9.63–11.44)		2.86 (0.90–9.10)	0.076	3.43 (0.77–15.35)	0.107
Hypertension		<0.001 *				
≤30	22.67% (13.79–33.79)		Reference	-	Reference	-
31–40	10.49% (9.33–11.75)		0.40 (0.23–0.70)	0.001 *	0.53 (0.25–1.10)	0.089
>40	12.77% (11.80–13.79)		0.50 (0.29–0.86)	0.013 *	0.58 (0.25–1.35)	0.206
Preeclampsia		0.029 *				
≤30	7.58% (2.51–16.80)		Reference	-	Reference	-
31–40	3.04% (2.39–3.81)		0.38 (0.15–0.98)	0.045 *	0.42 (0.12–1.42)	0.162
>40	4.04% (3.47–4.69)		0.51 (0.20–1.30)	0.158	0.48 (0.12–1.99)	0.314
PROM		0.016 *				
≤30	10.81% (4.78–20.20)		Reference	-	Reference	-
31–40	4.25% (3.50–5.11)		0.37 (0.17–0.78)	0.009 *	0.81 (0.22–3.00)	0.756
>40	4.06% (3.50–4.68)		0.35 (0.17–0.74)	0.006 *	1.15 (0.26–5.17)	0.851
Preterm birth		0.132				
≤30	15.92% (11.15–21.73)		Reference	-	Reference	-
31–40	11.64% (10.82–12.49)		0.70 (0.47–1.02)	0.065	0.64 (0.40–1.02)	0.063
>40	11.39% (10.78–12.02)		0.68 (0.46–1.00)	0.047 *	0.61 (0.35–1.06)	0.079
Delivery by cesarian section ^a^		<0.001 *				
≤30	47.31% (39.96–54.75)		Reference	-	Reference	-
31–40	50.95% (49.60–52.29)		1.16 (0.86–1.55)	0.33	0.63 (0.44–0.90)	0.012 *
>40	64.04% (63.06–65.00)		1.98 (1.48–2.65)	<0.001 *	0.61 (0.41–0.91)	0.017 *

Men were divided according to their age at conception (i.e., ≤30, 31–40, or >40 years old). Results are presented as a percentage with 95% confidence intervals (CI), odds ratio (OR) with 95% CI and *p* value of the comparison, and adjusted OR (AOR) and adjusted *p* value. PROM, premature rupture of membranes (prior to 37 weeks). * *p* < 0.05. ^a^ Proportion/probability of caesarean section (rather than vaginal) delivery.

**Table 3:** Perinatal outcomes associated with paternal age following in vitro fertilization or intracytoplasmic sperm injection using autologous sperm and donated oocytes.

	Proportion (%)/Mean (95% CI)	*p*	OR/RC (95% CI)	*p*	AOR/ARC (95% CI)	Adjusted *p* Value
Gestational age (days)		<0.001 *				
≤30	270.11 (267.39–272.84)		Reference	-	Reference	-
31–40	273.60 (273.18–274.02)		3.49 (1.23–5.74)	0.002 *	5.09 (2.39–7.79)	<0.001 *
>40	271.80 (271.50–272.11)		1.69 (−0.55–3.93)	0.139	4.54 (1.51–7.58)	0.003 *
Sex ^a^		0.11				
≤30	54.97% (47.63–62.16)		Reference	-	Reference	-
31–40	47.81% (46.47–49.15)		0.75 (0.56–1.00)	0.052	0.70 (0.49–0.99)	0.045 *
>40	48.72% (47.72–49.72)		0.78 (0.58–1.04)	0.088	0.75 (0.50–1.11)	0.153
Birth weight		0.535				
≤30	3118.60 (3010.19–3227.02)		Reference	-	Reference	-
31–40	3166.93 (3148.48–3185.38)		48.33 (−51.13–147.79)	0.341	63.75 (−25.13–152.64)	0.16
>40	3172.08 (3158.36–3185.80)		53.48 (−45.29–152.24)	0.289	81.96 (−18.31–182.23)	0.109
Low birth weight		0.279				
≤30	12.32% (7.34–18.99)		Reference	-	Reference	-
31–40	10.90% (9.95–11.90)		0.87 (0.52–1.46)	0.599	0.95 (0.41–2.19)	0.898
>40	10.04% (9.34–10.78)		0.79 (0.48–1.33)	0.38	0.92 (0.35–2.41)	0.865
Length at birth		0.010 *				
≤30	48.89 (48.16–49.63)		Reference	-	Reference	-
31–40	49.71 (49.62–49.81)		0.82 (0.27–1.37)	0.003 *	0.38 (−0.14–0.90)	0.156
>40	49.73 (49.66–49.80)		0.84 (0.30–1.39)	0.002 *	0.50 (−0.08–1.08)	0.09
Cranial perimeter		0.012 *				
≤30	34.00 (33.43–34.57)		Reference	-	Reference	-
31–40	34.38 (34.28–34.47)		0.38 (−0.17–0.92)	0.179	−0.02 (−0.55–0.52)	0.948
>40	34.52 (34.45–34.59)		0.52 (−0.02–1.06)	0.061	0.03 (−0.56–0.62)	0.922
Apgar score 1		0.256				
≤30	9.00 (8.65–9.35)		Reference	-	Reference	-
31–40	8.77 (8.71–8.83)		−0.23 (−0.56–0.09)	0.163	−0.33 (−0.70–0.05)	0.085
>40	8.81 (8.76–8.85)		−0.20(−0.52–0.13)	0.24	−0.28 (−0.69–0.14)	0.187
Apgar score 5		0.478				
≤30	9.72 (9.56–9.88)		Reference	-	Reference	-
31–40	9.60 (9.56–9.64)		−0.12 (−0.34–0.10)	0.28	−0.21 (−0.47–0.05)	0.107
>40	9.62 (9.59–9.64)		−0.11 (−0.32–0.11)	0.346	−0.20 (−0.48–0.08)	0.164
Apgar score 10		0.112				
≤30	9.75 (9.49–10.01)		Reference	-	Reference	-
31–40	9.76 (9.70–9.82)		0.01 (−0.29–0.30)	0.97	−0.21 (−0.55–0.13)	0.229
>40	9.82 (9.79–9.86)		0.07 (−0.22–0.37)	0.625	−0.20 (−0.57–0.18)	0.309
NICU admission		0.063				
≤30	19.54% (11.81–29.43)		Reference	-	Reference	-
31–40	11.55% (10.44–12.73)		0.54 (0.31–0.92)	0.025 *	0.74 (0.35–1.54)	0.418
>40	11.42% (10.60–12.28)		0.53 (0.31–0.91)	0.021 *	0.83 (0.36–1.93)	0.661

Men were divided according to their age at conception (i.e., ≤30, 31–40, or >40 years old). Results are presented as a proportion or mean with corresponding 95% confidence intervals (CI) and computed *p* values of the comparison between the three groups; odds ratio (OR) or regression coefficient (RC) with corresponding 95% CI and a *p* value of the comparisons, and adjusted OR (AOR) or adjusted RC (ARC) and adjusted *p* value. * *p* < 0.05. NICU, neonatal intensive care unit. ^a^ Percentage of live female births over the total number of live births.

## Data Availability

Not applicable.
